# Daily Mobility and Social Interactions Among Community-Dwelling Older Adults With Pet Dogs: A Scoping Review

**DOI:** 10.1177/07334648221116633

**Published:** 2022-08-26

**Authors:** Suellen Costa, Liliana Sousa, Helena Luz, Miguel Padeiro

**Affiliations:** 1 37829 University of Aveiro, Aveiro, Portugal; 2 CINTESIS@RISE, Department of Education and Psychology, 56062 University of Aveiro, Aveiro, Portugal; 3 University of Coimbra, Centre of Interdisciplinary Studies (CEIS20), Faculty of Psychology and Educational Sciences, Portugal; 4 Centre of Studies in Geography and Spatial Planning (CEGOT), 37829 University of Coimbra, Coimbra, Portugal

**Keywords:** companion dogs, older adults, social interaction, daily mobility

## Abstract

Dogs are part of many people’s lives and are involved in interventions to improve the well-being of older adults in institutional settings. However, the literature on the impact of pet dogs on community-dwelling older adults is still relatively limited. This study mapped the impact of having a companion dog on the daily mobility and social interactions of community-dwelling older adults using a scoping review. Electronic databases were searched, and studies written in English, Portuguese, and Spanish that were published in a peer-reviewed journal were identified. After a careful review, 26 eligible studies were identified, and relevant findings were extracted. The main findings indicated that having a dog may promote or hinder daily mobility and social interactions and that having a dog is about routines and sharing affection. More research is needed to clarify what makes having a companion dog key to promoting active and healthy aging.

**What this paper adds**

- Companion dogs are part of families and households and are relevant to individuals of all ages. Research has mainly focused on the role of therapy and service dogs in people’s lives. Dogs have been involved for therapeutic purposes in interventions targeting older adults with depression and dementia, have shown effectiveness mostly in institutional settings.

**Applications of study findings**

- Many older community-dwelling adults are dog owners, and it is important to better understand how companion dogs contribute to healthy and active aging. In this scoping review, the findings suggest that dogs contribute in terms of companionship and affection. However, in terms of daily mobility and social interactions, we observed mixed impacts that depend on the dog, owner, and environmental features.
- Older adult dog owners may spend more time at home to ensure that their dogs are not alone, thus limiting daily mobility and social interaction. Dog characteristics (size, age, and behavior) have been scarcely explored and may mediate dog–owner daily mobility and social interactions. Outdoor spaces, including dog-friendly spaces, increase the frequency and quality of activities outside the home, including with the companion dog.

## Introduction

For centuries, dogs have been part of human society as companion animals (Benz-Schwarzburg et al., 2020; Bergström et al., 2020). Dog companionship is prevalent worldwide. In Europe, dogs are the second most popular pets (cats are first), with about 24% of households owning at least one dog (The European Pet Food Industry, 2021), and in the US, about 54% of households own dogs (American Pet Products Association, 2019). Overall, pet dogs are a source of companionship that increases the health and well-being of their owners. The role played by pets in human aging is being increasingly explored (Bowen et al., 2021; Enders-Slegers & Hediger, 2019; Gee & Mueller, 2019; McCune et al., 2014). Research suggests that the human–animal relationship is linked to better mental health, reinforced social interaction, and the processes of physical, cognitive, and emotional rehabilitation, helping to maintain autonomy (Baun & Johnson, 2010; McCune & Promislow, 2021).

Active and healthy aging are paradigms boosted by the World Health Organization’s (WHO) proposal for a Decade on Healthy Ageing 2020–2030, in convergence with the United Nations 2030 Agenda for Sustainable Development (SDG 3), assuming healthy aging as a continuous process of optimizing functional ability that enables well-being in older age. Functional ability is determined by an individual’s physical and mental capacities in interaction with the individual’s environment. In this approach, functional capacities include the ability to meet basic needs, learn, grow, make decisions, contribute to society, be mobile, and build and maintain relationships (World Health Organization, 2020). Different environments, including the home, community, and broader society, are crucial for healthy aging because they are considered enablers of meaningful engagement among older people (World Health Organization, 2020).

As people age, mobility and opportunities to socially interact and form relationships tend to diminish. A set of factors contribute to that decrease: (i) the retirement process, which signals a decreased need for dislocation and loss of daily contact with co-workers (Handley et al., 2021); (ii) the health problems that often come as people age (Metz, 2000); (iii) physical frailty, which can affect mobility and contribute to a reduction in the number of social interactions (Gardner, 2014; Metz, 2000); and/or (iv) the mourning the loss of relatives, especially spouses and close friends (Prohaska et al., 2020). The lack of daily social interaction and reduced mobility are associated with social isolation, loneliness, and greater functional dependence (Guerra et al., 2021; Prohaska et al., 2020). Daily social interactions have the potential to enhance the well-being of older adults and improve their physical and mental health. Opportunities to build and/or maintain social networks are found in everyday activities, including walking dogs, which also potentiate mobility (Dall et al., 2017; Negrini, 2015). For older adults, walking within their communities can be a safe and easy way to stay physically active (Schmidt et al., 2019). Thus, promoting mobility within cities (e.g., walkability) can empower older adults (van Hoof et al., 2018), as urban areas constitute an invaluable resource in older people’s everyday lives by performing inspirational, social, and restorative functions (Negrini, 2015). Some studies have emphasized that having a pet, particularly a dog pet, has many benefits for older people, stressing that maintaining mobility and fostering social interaction are key aspects of a healthy aging process (Giannouli et al., 2019; Kojima et al., 2020). Indeed, dog walking encompasses a social component, since it may be an opportunity to socialize and increase the sense of community. Social interactions benefits may be a key component for walking the dog, in particular for more isolated or lonely older adults and their family caregivers (Christian et al., 2018).

The literature has examined the role of dogs in therapeutic purposes, such as assisted therapy, showing their contributions to all age groups. Specifically, dogs stimulate mobility, interpersonal contact, and communication (Rodrigo-Claverol et al., 2020). Dogs have been involved for therapeutic purposes in interventions targeting older adults with depression and dementia, mostly in institutional settings (Jain et al., 2020). However, literature on the impact of having a pet dog on community-dwelling older adults is still relatively limited. Therefore, this scoping review aims to map the impact of having a companion dog on the daily mobility and social interactions of community-dwelling older adults (≥65 years old).

## Research Design and Methods

This study adopted a scoping review approach following the stages developed by Arksey and O’Malley (2005) and updated by Levac et al., 2010. Scoping reviews map relevant evidence and identify gaps in a topic that are useful for emerging research areas. Data are reported following the checklist of the PRISMA-ScR (Tricco et al., 2016). A protocol was developed using the framework proposed by the Joanna Briggs Institute (Peters et al., 2020) and adjusted by the platform registration guidelines (Canellas et al., 2021). The final version is available INPLASY202190111 (Costa et al., 2021).

### Identifying the Research Question

The first author performed preliminary searches on aging and older adults, companion/pet dogs, daily mobility, and social interaction to refine the research question. Some full texts of selected articles were reviewed to understand how the terms have been used. Most studies reported on assistance or therapy dogs in institutional settings, which was not our focus but helped to clarify the terms. We identified the following research question: How does having a companion dog influence the daily mobility and social interactions of older adults (owners or guardians, aged ≥65 years old)?

### Identifying Relevant Studies

#### Searching Electronic Databases

This scoping review used a three-step search strategy to identify published articles. First, we used selected English search terms after analyzing the most frequently used keywords in articles published on Scopus within our research theme, which were tested in the indexed keywords from the Medical Subject Headings (MeSH) that are relevant to this review. Second, the university librarian verified the search strategies to adapt the keywords and index terms to each database requirement. References were searched through the following multidisciplinary and health-related databases: Scopus, Web of Science, PubMed, and Academic Research Completed. Third, the reference lists of articles identified during the search were manually checked to identify potential papers for inclusion.

Searches were conducted in June 2021. The search strategy included search terms (Table 1) related to the population, context, and concept (PCC). The population comprised community-dwelling older adults, aged ≥65 years, who had at least one companion dog. Our primary intention was to retain urban-based studies only; however, many studies based on nationwide and/or cohort data do not specify the type of settings. Removing them would unnecessarily reduce the total number of studies selected. Therefore, we included studies with at least part of the sample located in an urban environment. Location in either developed or emerging countries was not considered as an inclusion or exclusion criterion. Although strong differences between them could challenge this review’s results, the inclusion of countries regardless of their economic level would provide the possibility of addressing potential differences in the effects of dog ownership across a variety of situations. Older people were defined as those aged 65+ (developed countries) or 60+ (in emerging countries) as outlined by the World Health Organization (2020); however, we included other ages in cases where the studies identified the participants as older people.

**Table 1.** Search Terms and Inclusion and Exclusion Criteria.

Search Terms		
Population	Concept	Context
“older people” OR “older adult*” OR aging OR “old-age” OR age* OR ancient* OR elderl* OR senior* OR “later life” AND “dog* owner*” OR “dog tutor*” OR “companion dog*” OR “cane* familiar*” OR “domestic dog*”	“daily mobility” OR walk* OR mobility AND social* OR “social interaction” OR “interpersonal relationship”	community OR “community-dwelling”
Exclusion		
“service dog*” OR “guide dog*”	“animal-assisted interventions”	“therapeutic setting*” OR “therapeutic environment” OR “therapeutic residences” OR “residence for the older adult*” OR “residence for the elderl*”

Inclusion	Exclusion
All types of studies and reviews	Letters, commentaries, theses, books, gray literature, and abstracts published in proceedings
Focusing on older adults with a companion dog	Not focusing older adults dog owners
Studies including (not necessarily exclusively) urban community dwellings	Addressing animal-assisted intervention and/or aged care facilities and/or involving guide or therapeutic dogs
Addressing daily mobility and/or social interactions	Articles that are inaccessible

#### Screening

The inclusion and exclusion criteria applied during the two screening stages (titles and abstracts, as well as full text) are shown in Table 1. First, the titles and abstracts were selected by two independent reviewers (first and second authors). Data from each relevant publication were imported into the reference management software (Mendeley 1.19.8). Second, the same program was used to delete duplicates. Third, the first author exported the titles and abstracts of the selected articles into a spreadsheet (Excel, 2016) to identify the studies to be excluded. The second author independently did the same. Disagreements were resolved through discussion with the remaining authors. Fourth, the full texts of the selected articles were obtained and read by the first author. Any disagreement was addressed through discussions with another author.

#### Selecting Studies and Charting the Data

**Figure 1.**
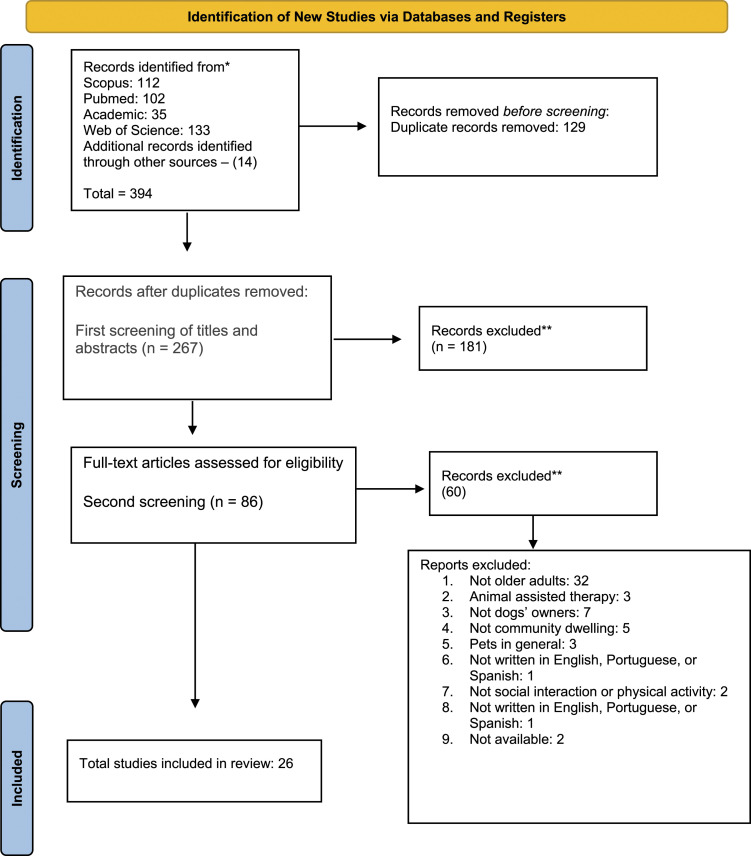
Flow diagram for the scoping review process.

Eighty-six studies were initially identified for screening. Afterward, 26 remained (Figure 1). The main criterion for exclusion was not focusing on older adults; most of these studies included older adults in the samples, but the data analysis did not differ by age group. The following variables were extracted: first author, year of publication and country, objective(s), geographical context, methodology/design, sample, instruments and indicators, daily mobility, social interactions, dog-related variables, environmental variables, main findings, and other variables. The data were extracted into Microsoft Excel. Data analysis followed a descriptive form to map evidence according to the review question; the main findings were addressed through a narrative review (Peters et al., 2020). For that end, the rules to create categories based on the aforementioned characteristics were first established. The authors then discussed how to allocate the findings to the different categories, and characteristics were counted within each category. The findings were then described based on the categorization, and a synthesis regarding daily mobility and social interaction major findings was produced.

## Results

### Overview of Selected Studies

Table 2 offers a general overview of the included studies (years, geographic context, methods and design, and samples). The 26 included papers were published between 1993 and 2021, with a large publication gap between 1993 and 2006 (Table 2). Most of the papers (*n* = 15) were published from 2017 onwards. The geographical origins are as follows: 42.3% came from North American countries (US: 10; Canada: 1) and 38.5% came from European countries (UK: 4; Austria: 1; Bulgaria: 1; Czech Republic: 1, The Netherlands: 1; Spain: 1). Three other papers (11.5%) were from Japan (2) and China (1). The remaining one was from Australia.

**Table 2.** General Data on Studies Selected.

Studies	Geographic Context	Methods/Design	Sample
Arbillaga-Etxarri et al. (2017, Spain)	Five cities of Catalunya	QT. Cross-sectional; ANOVA, Kruskal–Wallis, and Wilcoxon rank-sum tests, multivariable regression	COPD patients (chronic obstructive pulmonary disease); *N* = 410; average age: 69; male: 85%
Carr et al. (2021, USA)	Florida (no details)	QT. Longitudinal; OLS regression models	Community adults aged 60+; *N* = 466; average age: 69.4; male: 34%; dog owners: 39–42%
Chen et al. (2020, China)	Urban; Guangzhou	QL. Participatory observations; in-depth interviews	Urban empty nesters; *N* = 12; average age: 66.7; male: 25%; dog owners: 100%
Curl et al. (2017, USA)	USA (no details, nationwide survey)	QT. Cross-sectional; OLS regression and binary logistic regression models	Community-based adults aged 50+; *N* = 771; average age: 67.7; male: 48.8%; dog owners: 35%
Curl et al. (2020, USA)	USA (no details, nationwide survey)	QT. Cross-sectional; structural equation modeling (SEM)	People aged 50+; *N* = 476; average age: 68.97; dog owners: 39%
Dall et al. (2017, The Netherlands)	Lincolnshire, Derbyshire, Cambridgeshire (no details)	QT. Generalized linear mixed-effect model	People aged 65+; *N* = 86; average age: 70; male: 34%, dog owners: 50%
Dzhambov (2017, Bulgaria)	Plovdiv, urban area near green areas	QT. Cross-sectional; correlations and mediation analysis	Urban community-dwelling retired people aged 65+; *N* = 265; average age: 75.8; male: 46.4%; dog owners: 100%
Feng et al. (2014, UK)	Tayside; all types of areas	QT. Cross-sectional, chi-square, independent sample t-tests, and mixed linear models	Community-dwelling people aged 65+; *N* = 547; average age: 79; male: 46%; dog owners: 9%
Friedmann et al. (2020, USA)	Baltimore (no details)	QT. Cross-sectional; ANOVA, linear regression	Community-dwelling people aged 50+; *N* = 378; average age: 76.9; male: 43%; dog owners: 24%
Garcia et al. (2015, USA)	USA (no details, nationwide survey)	QT. Cross-sectional; logistic regression	Postmenopausal women aged 50 to 79; *N* = 152,629; average age: 61.5; dog owners: 24%
Gretebeck et al. (2013, USA)	USA (no details)	QT. Cross-sectional; ANOVA, hierarchical multiple regressions	Community-dwelling older adults aged 65+; *N* = 1091; average age = 74.5; male: 44%; dog owners: 15%
Harris et al. (2009, UK)	Oxfordshire (no details)	QT. Cross-sectional; linear regression models	Community-dwelling older adults; *N* = 238; male: 52%; dog owners: 22%
Hui Gan et al. (2020, Australia)	Not stated	QL. Phenomenological; semi-structured interviews	Community-dwelling older adult; *N* = 14; average age: 73.6; male: 43%; dog owners: 100%
Janevic et al. (2020, USA)	USA (no details)	QL. Focus groups; thematic analysis	Community-dwelling older adults aged 70+; *N* = 25; male: 32%; dog owners: 72%
Koohsari et al. (2021, Japan)	Predominantly rural, Minami-Izu	QT. Cross-sectional; linear regression models	Young, middle-aged and older adults; *N* (≥65) = 1722; male: 45%; dog owners: 12%
Mein and Grant (2018, UK)	Urban, London	QT. Cross-sectional; multivariable proportional odds regression	People aged 59–79; *N* = 6575; average age: 65.1; dog owners: 11%
Mičkova (2019, Czech Republic)	Czech Republic (no details)	QT. Cross-sectional; Spearman rank correlation	People aged 60+; *N* = 44; average age: 68; male: 41%; dog owners: 59%
Moniruzzaman et al. (2015, Canada)	Urban and suburban; Metro Vancouver	QT. Cross-sectional; multilevel linear regression	Low-income older adults aged 65+; *N* = 145; male: 35%; dog owners: 10%
Rijken (2010, Netherlands)	Nationwide	QT. Cross-sectional; linear and logistic regression models	People aged 65+; *N* = 1397; male: 40%; dog owners: 10%
Rogers et al. (1993, USA)	Mobile home parks in Sacramento	QT + QL. Walk (with and without dogs), interview, quantitative comparisons, and content analysis	People aged 65+; *N* = 11; average age: 69; male: 27%; dog owners: 55%
Scheibeck et al. (2011, Austria)	Larger communities of the Tyrol (no details)	QL. Systematic literature research, ethnographic field	People aged 70+; *N* = 23; male: 22%; dog owners: 100%
Shibata et al. (2012, Japan)	Urban; Bunkyo, Fuchu, Oyama	QT. Cross-sectional; multivariate logistic regression models	Community-dwelling older adults aged 65–74; *N* = 1926; average age: 69.6; male: 51%; dog owners: 14%
Taniguchi et al. (2018, Japan)	Urban; Ota City, Tokyo	QT. Cross-sectional; mixed-effects logistic regression models	Community-dwelling older adults aged 65+; *N* = 11,233; male: 48%; dog owners: 14% (past owners: 29%)
Thorpe Kreisle et al. (2006, USA)	Memphis, TN, and Pittsburgh, PA (no details)	QT. Cross-sectional (part of a cohort study); chi-square, t-tests, and logistic regression models	Community-dwelling older adults aged 65+: *N* = 2533; male: 52%; dog owners: 24%
Thorpe Simonsick et al. (2006, USA)	Memphis, TN, and Pittsburgh, PA (no details)	QT. Cross-sectional and longitudinal; general linear models, and logistic regression models	Community-dwelling older adults aged 65+; *N* = 2533; male: 52%; dog owners: 24%
Wu et al. (2017, UK)	Norfolk County	QT. Cross-sectional; multilevel first-order autoregressive models	Adults aged 40–79 (73% aged 65+); *N* = 3123; male: 43%; dog owners: 18%

Studies conducted in urban settings account for 46% of the studies selected, while studies explicitly including urban and rural areas represent 12% (Feng et al., 2014; Koohsari et al., 2021; Wu et al., 2017). The remaining 42% do not provide details about the urban or rural environments, but presumably include both, as they are state-wide, nationwide studies, or conducted in counties or regions including both rural and urban areas.

The methods were mostly quantitative (*n* = 21), with four qualitative studies (Chen et al., 2020; Hui Gan et al., 2020; Janevic et al., 2020; Scheibeck et al., 2011) and one using mixed approaches (Rogers et al., 1993). Among the quantitative studies, most used a cross-sectional design (*n* = 19), and two longitudinal approaches were used (Carr et al., 2021; Dall et al., 2017). Sample sizes ranged from 11 to 152,629 participants: 44–152,629 participants in quantitative; 12–25 participants in qualitative. The study (Rogers et al., 1993), using both approaches, had 11 participants. The minimum age in most studies was 65 (*n* = 14), while two studies included participants aged ≥70 (Janevic et al., 2020; Scheibeck et al., 2011). One study did not indicate a lower limit (Arbillaga-Etxarri et al., 2017). In five studies, the age limit was 50 years; in three studies, it was 60 years. One study considered 49 years to be the lower limit (Wu et al., 2017); no distinction could be made between them the age groups in terms of results. The average age was above 70 in six studies and under that in nine studies. In four studies, the non-dog owners group was older than the dog owners group, with differences ranging between 1.3 and 6 years (Curl et al., 2017; Garcia et al., 2015; Gretebeck et al., 2013; Mein & Grant, 2018). Two studies covered the adult population (≥18 years old), but both analyzed the sample of older people (≥65 years old). Women were predominant (above 51%) in the samples of 18 studies.

### Objectives and Instruments

Objectives and instruments used to assess the outcomes are shown in Supplementary Table 1. Regarding the study’s objectives, 16 examined daily mobility, three focused on social interactions, and seven included both dimensions. Instruments widely varied. Most studies comprising daily mobility analyzed levels of physical activity and/or walking behavior. One study focused on mobility patterns beyond walking and included car- and public transit-related mobility (Moniruzzaman et al., 2015). Studies including social interaction examined the frequency of social contact and neighborhood engagement (Curl et al., 2020; Mein & Grant, 2018). In addition, five studies investigated mental well-being: two examined loneliness (Carr et al., 2021; Rijken & van Beek, 2011), three explored dimensions of mental well-being (Chen et al., 2020; Curl et al., 2020; Hui Gan et al., 2020), and one examined pain management (Janevic et al., 2020).

### Explanatory Variables

The studies included a variety of potentially contributing factors (Supplementary Table 2). Nine studies included environmental variables as potential moderators of the relationship between dog-related variables and outcomes (Table 3). These included neighborhood deprivation (Arbillaga-Etxarri et al., 2017), neighborhood general perceptions (Dzhambov, 2017; Moniruzzaman et al., 2015), neighborhood greenness and/or the quality of public space for dog walking (Arbillaga-Etxarri et al., 2017; Chen et al., 2020; Dzhambov, 2017; Feng et al., 2014), having a backyard (Scheibeck et al., 2011), weather conditions (Feng et al., 2014; Wu et al., 2017), or distinctions between study sites (Thorpe, Kreisle, et al., 2006; Thorpe, Simonsick, et al., 2006).

**Table 3.** Main Findings.

Studies	Daily Mobility	Social Interaction	Effect on Daily Mobility?	Effect on Social Interaction?
Arbillaga-Etxarri et al. (2017, Spain)	Dog walking was significantly associated with an increase in time in MVPA and in physical activity intensity. Neighborhood deprivation, surrounding greenery, and proximity to green or blue spaces were not associated with physical activity	None	Positive	—
Carr et al. (2021, USA)	None	Walking a dog at least once a day offset increases in loneliness among older adults who experienced significant social consequences related to COVID-19	—	Positive
Chen et al. (2020, China)	Outdoor activities can satisfy the needs of companion dogs and increase exercise levels for urban empty nesters. Companion dogs share the same rituals and rhythms of owners, and they motivate each other to reach a state of self-discipline. Companion dogs motivate owners to overcome mental and physical challenges. For empty nesters, it is an opportunity to take care of others	None	Positive	—
Curl et al. (2017 USA)	Owning a dog indicated an average effect of 22 min of additional time walking and 2760 additional steps per day. Dog owners had significantly fewer sitting events. There were no differences between the groups in the total time spent sitting, number, or duration of sedentary events	None	Mixed	—
Curl et al. (2020, USA)	None	Time spent dog walking was associated with the frequency of social interactions. Bond with a dog was associated with dog walking. There were no differences between dog owners and non-pet owners in terms of social contact	—	Mixed
Dall et al. (2017, The Netherlands)	Better park quality was related to less dog walking time and to poorer perceived health; more visitors attracted increased complaints	None	Mixed	—
Dzhambov (2017, Bulgaria)	Dog walking was associated with lower BMI, fewer activities of daily living limitations, fewer doctors’ visits, and more frequent moderate and vigorous exercise. Dog ownership was not associated with better physical health or health behaviors	None	Mixed	—
Feng et al. (2014, UK)	Dog walkers reported more minutes/week of moderate-to-vigorous physical activity and total physical activity than non-dog walkers and non-dog owners	None	Positive	—
Friedmann et al. (2020, USA)	Dog ownership predicted higher levels of daily energy expenditure. Dog owners who walked their dogs reported walking at about the same speed or slower than when they walked without the dog	Negative effects of pet ownership occurred infrequently, and positive influences occurred frequently. Dogs were more likely (36.0%) than cats (12.0%) to facilitate social interaction and to cause owners to decline visits with family members	Positive	Mixed
Garcia et al. (2015, USA)	Dog ownership was positively related to higher physical activity levels. Dog owners were 12% more active than non-dog owners	None	Positive	
Gretebeck et al. (2013, USA)	Dog owners walked quite a distance each day, and they were in general physically healthy	None	Positive	—
Harris et al. (2009, UK)	Dog owners were more likely than non-pet owners to have engaged in non-exercise-related walking; this did not differ from non-pet owners in walking for physical activity. The activity-related benefits of pet ownership were limited to dog owners who engaged in greater physical activity, particularly non-exercise-related walking	None	Mixed	—
Hui Gan et al. (2020, Australia)	Pets were a source of motivation to engage older adults in activities. Pets played an important role in enabling them to have something productive to look forward to, giving owners a sense of purpose and value	Pet ownership meant engagement in pet-related activities resulting in increased socialization with friends and family, which provided a sense of belonging to the community. The pet was viewed as a “connector”	Positive	Positive
Janevic et al. (2020, USA)	Pets provided motivation for physical activity and offered no choice. Pet ownership requires adherence to a routine. The potential negative effects of physical activity with pets include (fear of) injury due to a rambunctious dog or strain from a heavy pet	Having pets increased social activity with people, helping to build or maintain relationships. However, pets may have a negative impact on social activity due to certain behaviors	Mixed	Mixed
Koohsari et al. (2021, Japan)	None	There were no differences in the means of social capital between the three groups (non-dog owners, dog owner non-walkers, and dog owner walkers). There was no link between dog walking and social cohesion	—	No effect
Mein and Grant (2018, UK)	Mild exercise in terms of metabolic equivalents and moderate exercise were higher in pet owners than non-owners and in dog owners than owners of other types of pets. There were no differences in terms of vigorous exercise	Pet owners were more positive about their neighborhood than non-owners	Mixed	Positive
Mičkova (2019, Czech Republic)	There were differences in favor of dog owners in most of the monitored parameters, specifically higher total physical activity time (min/week), MET/min/week spent in walking, and spent calories/week	None	Positive	—
Moniruzzaman et al. (2015, Canada)	Dog owners/dog walkers reported a significantly higher walking, walking frequency, leisure and physical activity level, as well as total functional ability, than non-dog walkers or owners. Pet obligations may provide purposeful activities that motivate some older dog owners to walk	None	Positive	—
Rijken (2010, Netherlands)	Dog walkers were more likely to achieve 150 minutes of walking per week and had faster usual and rapid walking speeds. Three years later, dog walkers experienced similar declines in usual and rapid walking speed as non-dog owners, but maintained their initial mobility advantage	None	Positive	—
Rogers et al. (1993, USA)	Dog owners reported a pattern of walking twice a day, whereas non-owners’ reported a walk of only once a day	Dog owners and non-owners had good social interaction. Dog owners reported more satisfaction with social, physical, and emotional states	Positive	Positive
Scheibeck et al. (2011, Austria)	Activities associated independently with higher step counts included the number of long walks and dog walking. The strongest associations were with the number of long walks and dog walking	None	Positive	—
Shibata et al. (2012, Japan)	Older adults living with a dog were healthier and more active than the group of non-owners or the groups of cat or other pet type owners	No associations between pet ownership and the frequency of social contacts or feelings of loneliness	Positive	No effect
Taniguchi et al. (2018, Japan)	Physical activity showed a significant association with dog ownership	Social function showed a significant association with dog ownership	Positive	Positive
Thorpe Kreisle et al. (2006, USA)	Dog ownership was associated with reduced trip distance. The interaction between dog ownership and walking as a transportation mode had a positive association with trip distance. Dog owners assumed the responsibility to walk their dogs independent of the built environment	None	Mixed	—
Thorpe Simonsick et al. (2006, USA)	Total minutes of walking was not different between dog owners and non-dog owners. Owning a dog was associated with a higher likelihood of walking 150 min/week and a lower chance of being sedentary	None	Mixed	—
Wu et al. (2017, UK)	Regular dog walkers were more active on days with the poorest conditions than non-dog owners were on the days with the best conditions	None	Positive	—

In 21 studies, dog ownership was included as an explanatory variable. In four qualitative studies, 100% of the participants had dogs. Pet bonding was a potential explanatory variable in three studies, and two included information about the dog, such as age or size.

Some additional variables were considered (Supplementary Table 1). First, having a dog does not necessarily mean walking the dog, since not all dog owners take their dogs for a walk (Curl et al., 2017). Many people have a fenced yard where the dog can practice physical activity, while others have physical limitations that prevent them from walking the dog. Second, higher levels of pet bonding seem to be associated with more time spent dog walking (Curl et al., 2017, 2020) and to a better perception of the neighborhood (Mein & Grant, 2018). Scheibeck et al. (2011) found that responsibility and attachment to the dog give owners a sense of purpose that comes from routine, with fixed times for meals and walks. Carr et al. (2021) showed that the attachment to the dog was a motivation to remain physically active to meet the animal’s needs. Third, age may moderate the relationship between dog ownership, mobility, and social interaction. Dog ownership tends to decrease with age (Curl et al., 2017; Garcia et al., 2015; Gretebeck et al., 2013; Mein & Grant, 2018; Wu et al., 2017). Regarding social interactions, Koohsari et al. (2021) did not find significant associations between dog ownership and activities with neighbors, contrary to other age groups. Mein and Grant (2018) reported that age is not a moderator in the relationship between dog ownership and social activities, while Chen et al. (2020) found that having a dog increases mobility and social interaction according to age.

The quality of the outdoor residential environment is a contributing factor to daily mobility and social interaction. The quality and walkability of the built environment (perceived or objective) and the presence and/or number of green areas close to home (Chen et al., 2020) encourage people to enjoy public spaces and social contact. The existence of dog-friendly outdoor spaces (especially in contexts with strong regulations that ban dogs in certain places) allows for an increase in the frequency and quality of activities conducted outside the home (Chen et al., 2020). However, this is not always the case. Arbillaga-Etxarri et al. (2017) found that for older people with chronic obstructive pulmonary disease (COPD), the characteristics of the built environment related to green or blue spaces around homes were not associated with physical activity levels. Dzhambov (2017) reported that better park quality can be related to less dog walking time by older adults due to more visitors being attracted and increasing the number of complaints regarding dog behavior (not behaving well on a leash) from other users (in particular, younger owners).

### Synthesis of the Findings

Table 3 provides the main findings of the included studies.

#### Daily Mobility

A total of 23 studies addressed daily mobility as an outcome; 15 found a positive relationship arising from dog ownership (Table 3). No study found an exclusively negative relationship. These studies showed that dog ownership is associated with higher levels of physical activity, measured through the frequency of dog walking (Arbillaga-Etxarri et al., 2017; Friedmann et al., 2020; Gretebeck et al., 2013), the total time spent dog walking (Arbillaga-Etxarri et al., 2017; Gretebeck et al., 2013; Harris et al., 2009; Shibata et al., 2012; Thorpe, Simonsick, et al., 2006), distances walked even without a dog (Moniruzzaman et al., 2015), or the intensity of physical activity (Arbillaga-Etxarri et al., 2017; Feng et al., 2014; Friedmann et al., 2020; Gretebeck et al., 2013; Shibata et al., 2012; Wu et al., 2017). Other studies showed that dog ownership forces people to adopt a routine that is difficult to escape (Chen et al., 2020; Hui Gan et al., 2020; Janevic et al., 2020), representing an additional motivation to leave the house and walk, even in adverse weather conditions (Wu et al., 2017). This stimulation provides a sense of purpose (Hui Gan et al., 2020). A qualitative study (Janevic et al., 2020) showed that the relationship between dog ownership and health was generally positive but mentioned some negative aspects, such as “injury or fear of injury due to walking a rambunctious dog, or strain from picking up a heavy pet” (p. 1092). Six studies found mixed results (Curl et al., 2017; Dall et al., 2017; Garcia et al., 2015; Mein & Grant, 2018; Moniruzzaman et al., 2015; Thorpe, Kreisle, et al., 2006). Garcia et al. (2015) found no difference between dog owners and non-dog owners in terms of the total minutes spent walking. Dall et al. (2017) showed no significant differences between dog owners and non-dog owners in terms of sedentary time. Curl et al. (2017) showed that dog ownership is not associated with better physical health or health behaviors. The absence of positive effects is related to the speed of walking and the distance covered, which are frequently lower in older adults with a dog compared to those who walk without a dog (Curl et al., 2017; Friedmann et al., 2020; Rogers et al., 1993), which means a lower tendency toward more vigorous physical activity.

#### Social Interaction

Ten studies addressed social interactions as an outcome, and eight found a positive relationship between dog ownership and social interactions (Table 3). Globally, having a dog encourages the involvement of older adults in social activities in the neighborhood, increases socialization with friends and family (Friedmann et al., 2020; Hui Gan et al., 2020; Janevic et al., 2020; Mein & Grant, 2018, increases social interaction in public spaces (Taniguchi et al., 2018), and is associated with better perceptions of the neighborhood (Mein & Grant, 2018). A study conducted in the context of the COVID-19 pandemic showed that dog walking was associated with less increase in loneliness (Carr et al., 2021). Two studies pointed out that having a dog may have negative effects on social interactions (Friedmann et al., 2020; Janevic et al., 2020); they suggested that owners may decline visits with family members due to concerns regarding the well-being of the dog during their absence. Three studies showed mixed to no effects of having a dog (Koohsari et al., 2021; Rijken & van Beek, 2011). In addition, social contact may depend more on time spent dog walking than on having a dog (Curl et al., 2020).

## Discussion

This scoping review was performed to map the impact of having a companion dog on the daily mobility and social interactions of community-dwelling older adults. Overall, the results showed that (i) having a dog may promote or hinder daily mobility and social interaction and (ii) having a dog is about routines and sharing affection. This scoping review allowed for the identification of gaps in research, particularly the overlooked role of dog characteristics and the local environment.

### Having a Dog May Be Promoting or Hindering Daily Mobility and Social Interactions

Regarding daily mobility, the findings showed that having a dog did not correlate with walking the dog or engaging in some kind of physical activity with the dog. However, most studies did not distinguish dog owners who walk from those who do not walk their dogs. For these studies, the results tended to show gains in overall daily mobility and associated health conditions. Studies also showed that dog owners who walk the dog may be walking decreased distances and/or for less time since they are with the dog. Dog owners (even those who do not walk the dog) may spend more time at home to avoid leaving the dog alone.

Regarding social interaction, the studies pointed out that older dog owners had an increased opportunity for contact and social interaction with new people and neighbors, particularly when walking the dog. Establishing social and support networks for dog owners contribute to their satisfaction with life (Curl et al., 2020) and ability to overcome loneliness (Ikeuchi et al., 2021). Participation in neighborhood activities arising from dog ownership (Hui Gan et al., 2020; Mein & Grant, 2018; Taniguchi et al., 2018) can be seen as an opportunity for community participation and involvement that enriches the sense of community and strengthens the social bonds and social capital of older adults. These are key elements of healthy aging (Feng et al., 2014; Hui Gan et al., 2020; Koohsari et al., 2021) and reinforce that dogs can act as catalysts for social interactions (Christian et al., 2018). Some studies have reported that companion dogs reduce feelings of isolation and loneliness by acting as means of socio-emotional support for owners (not by facilitating interactions with other individuals). However, some have reported that dog owners may limit their interactions with relatives and friends to stay at home with the dog (Friedmann et al., 2020; Janevic et al., 2020). Others have reported no effect of dog ownership on the frequency of social contacts (Curl et al., 2020; Shibata et al., 2012), on feelings of loneliness (Shibata et al., 2012), or on social capital (Koohsari et al., 2021).

### Having a Dog Is About Routines and Sharing Affection

Findings suggested the importance of routine and responsibilities with dogs (e.g., walking, care with food, and leisure) as practices and positive motivation to keep older adults engaged and participating in society (Curl et al., 2017; Hui Gan et al., 2020; Janevic et al., 2020). This is relevant for active and healthy aging, namely, to the maintenance of the functional abilities and health among older people (World Health Organization, 2020). Recent research (McCune & Promislow, 2021) has stressed the need to focus on the pet’s role in creating healthier and more engaged communities. Increased movement (Gretebeck et al., 2013; Taniguchi et al., 2018) associated with a companion dog can create a positive impact by reducing the limitations of daily life. Companion dogs are a key element in the sharing of affection. Dogs occupy a space of companionship and emotional support to the point of replacing the owner’s absence of social interactions (Ikeuchi et al., 2021). Dotson and Hyatt (2008) addressed the issue of anthropomorphizing that occurs among dog owners. Scheibeck et al. (2011) pointed out the relationship between owners and their deceased dogs, describing the rituals of mourning and tomb ornaments like those made for humans. Rogers et al. (1993) noted that on dog walks, owners communicate with dogs in the same way they communicate with children. When they meet other people, the subject is usually about the dog. These data strengthen the conceptions (Souza Cabral & Savalli, 2020) that the socio-emotional support that dogs provide to owners, especially in Western society, satisfies the human need for affection and strengthens the companionship and love acquired from having a dog.

### Identified Gaps

#### The Overlooked Role of the Local Environment

Dzhambov (2017) considered environmental aspects to characterize the preferences and frequency of use of parks by older adult dog owners and found that reduced mobility due to age and generational difficulties might cause older adults to use less structured spaces and more isolated parks. Specifically, regarding accessibility to transport as an environmental aspect, Moniruzzaman et al. (2015) analyzed travel behavior among low-income older adults. The geographic context was shown to be an important factor in the perceptions of mobility. These contextual factors included the infrastructure, walkability, and accessibility levels of cities where older dog owners live. Assuming that mobility factors, the residences of individuals, the cities in which h they live, and the services provided were found to be transversally related to mobility, and they can act as enhancers or inhibitors. Further research could address the role of the local environment.

#### The Overlooked Role of Dog(s) Characteristics and Number

Most studies did not address variables related to dog characteristics. A few studies suggested that dog size, age, and behavior may influence the dog owner’s daily mobility and social interaction. For instance, an old dog may prefer to stay at home or go on short walks; an older person may find it difficult to manage a big dog, especially if the dog has more difficult behavior (hard to walk on a leash, aggressive, or too playful). Only three studies mentioned participants owning more than one dog (Dall et al., 2017; Friedmann et al., 2020; Hui Gan et al., 2020), and none of them provided information about a potential effect either on social interactions or on daily mobility. Recent surveys suggest that multiple pet’s ownership concerns more than one third of total households owning pets (Applebaum et al., 2021). Samples in the studies by Friedmann et al. (2020) and Dall et al. (2017) included 25 and 28% of multi-dog ownership respectively. Multiple dogs’ ownership may be associated to more intense or frequent activity due to increased needs, but increased constraints may reduce frequency of social contacts and increase difficulty in multiple dog walking and the risk of accidents. Both variables thus need to be investigated in future research.

#### Limitations

This review is not without some limitations. First, this review only included dogs as companion animals. The existence of other companion animals (in addition to the dog or as an exclusive pet) could modify the findings. It is likely that cat (or other animal) owners experience their local environment differently and that their degree of freedom in terms of daily mobility, outdoor time duration, and social interaction differs from that of dog owners. Our findings may not extend to other companion animals. Second, we focused on the impacts on daily mobility and social interaction and omitted other possible impacts, such as family interactions and relationships. Our focus was on older adults’ everyday lives in the community. However, it is possible that even family interactions, ties, and functions may be (positively or negatively) affected by dog ownership. In addition, we did not include studies that focused on more general health behaviors, such as the utilization of local and health services, a healthy diet, or even physical activity other than walking. Third, the inclusion of studies focusing on urban and rural areas without providing enough details, instead of limiting the geographical scope to exclusively urban settings, is another limitation. Such a limitation is an additional argument in favor of paying more attention to environmental variables in future research. Fourth, as in most scoping reviews, selection bias may have arisen from the restrictions imposed on the searches. Given that the selection criteria limited articles to those published in English, Portuguese, and Spanish, the literature reviewed might not be inclusive, and studies conducted in other languages were missed. Nevertheless, this scoping review highlights the importance of companion dogs for community-dwelling older adults.

## Conclusion

While research on older adults and companion dogs is still relatively limited, the topic has been receiving more attention in recent years. Having a companion dog provides community-dwelling older adults with companionship and a routine that motivates them and gives them a purpose. This is why better environments and more opportunities should enable them to enjoy the company of their dogs. However, companion dogs may either hinder or promote the daily mobility and social interactions of older adults. More research is needed to clarify what makes having a dog a key variable in promoting active and healthy aging.

## Supplemental Material

Supplemental Material - Daily Mobility and Social Interactions Among Community-Dwelling Older Adults With Pet Dogs: A Scoping Review Click here for additional data file. Supplemental Material for Daily Mobility and Social Interactions Among Community-Dwelling Older Adults With Pet Dogs: A Scoping Review by Suellen Costa, Liliana Sousa, Helena Luz, and Miguel Padeiro in Journal of Applied Gerontology
