# Supplementary material for: Daily Mobility and Social Interactions Among Community-Dwelling Older Adults With Pet Dogs: A Scoping Review
Source: J Appl Gerontol. 2022 Aug 26;41(12):2609–23. doi: 10.1177/07334648221116633 (PMC9669735; doi:10.1177/07334648221116633)
Supplement: Supplemental Material - Daily Mobility and Social Interactions Among Community-Dwelling Older Adults With Pet Dogs: A Scoping Review [file sj-pdf-1-jag-10.1177_07334648221116633.pdf]

Supplementary Table 1. Objectives and measurements.

Studies	Objective	Instruments/indicators: daily mobility	Instruments/indicators: social interaction
Arbillaga-Etxarri (2017, Spain)	Assess the relationship between socio-environmental factors, namely dog walking, grandparenting, neighborhood deprivation, residential surrounding greenness, residential proximity to green or blue spaces, and amount and intensity of physical activity in COPD patients.	Time spent on moderate-to-vigorous physical activity (min/day), magnitude units per minute; steps per day, walking time, time spent on physical activity.	None.
Carr (2021, USA)	Evaluate the potential benefits of dog walking in buffering the social consequences of COVID-19 on loneliness.	None.	Social cohesion, activities with neighbors.
Chen (2020, China)	Assess the association between dog ownership and the reconstruction of the later life of older empty nesters.	None.	Loneliness (based on a modified version of the UCLA Loneliness Scale).
Curl (2017, USA)	Explore the associations between dog ownership and pet bonding with walking behavior and health outcomes in older adults.	Frequency and time of dog walking; frequency, speed, distance with and without dog.	None.
Curl (2020, USA)	Examine the relationships between dog ownership, dog walking, emotional bond with a dog to neighborhood engagement, and life satisfaction.	Daily outdoor activity.	None.
Dall (The Netherlands, 2017)	Evaluate the influence of dog ownership on health-enhancing physical activity and sedentary behavior among community-dwelling older adults.	Frequency of dog-walking (days per week), Time of dog-walking (per week).	None.
Dzhambov (2017, Bulgaria)	Assess whether the effect of park quality on the time spent by seniors walking their dogs is mediated by the attitude of other park visitors toward the dogs and influences dog guardians' perceived health.	Time spent walking, time spent walking with a cadence of over 100 steps/min (equivalent to MVPA [23]), number of steps taken and time spent standing.	None.
Feng (2014, UK)	Examine whether dog ownership amongst community-dwelling older adults (≥ 65 years) is associated with objectively measured physical activity.	Minutes/week of total physical activity; Moderate- to vigorous-intensity physical activity (MVPA).	None.
Friedmann (2020, USA)	Examine the relationship between pet ownership and cognitive and physical functions and psychological status among community-dwelling older adults.	None.	Average number of social contacts per week with neighbors to chat or for a social visit and the unit of time (e.g., week, month; 7×/week and 30×/).
Garcia (2015, USA)	Examine cross-sectional associations between dog ownership and physical activity measures in a well-characterized, diverse sample of postmenopausal women.	Current and past experiences of physical activity: London Health and Fitness Questionnaire.	None.
Gretebeck (2013, US)	Identify factors that influence older adult walking and compare physical activity, functional ability, and psychosocial characteristics according to dog ownership status.	Frequency, distance, and duration of dog walking.	None.
Harris (2009, UK)	Examine the associations between physical activity levels and health, disability, anthropometric measures, and psychosocial factors.	Walking for exercise, non-exercise-related walking, vigorous activity (frequency and time).	None.
Hui Gan (2020, Australia)	Explore the influence of pet ownership on mental health among community-dwelling older adults.	Physical function (gait speed, daily activity level), time for each walk, number of calories of activity per day.	5-point Likert scale: (1) declined to visit out of concern for their pet's welfare; (5) found that having a pet(s) encourages them to be more

			socially active.
Janevic (2020, USA)	Explore how pet ownership promotes the use of pain self-management strategies among community-dwelling older adults.	Questions on mental health benefits of owning pets, the influence of pets on a daily routine.	
Koohsari (2021, Japan)	Estimate the differences in social capital by dog ownership and dog walking status among young-to-middle-aged adults and older adults.	How pets affected their health, including psychological functioning, pain, fatigue, and physical and social activity; facilitators, barriers, and concerns about pet care.	
Mein (2018, UK)	Explore associations between dog ownership and sleep, health, exercise, and neighborhood.	Physical function and activity (mobility limitation, body mass index, Motor Fitness Scale, physical activity, and frailty status).	Social function (interaction with neighbors, social isolation, trust in neighbors, frequency of going outdoors).
Mičkova (2019, Czech Republic)	Evaluate the effect of dog ownership on PA in older adults.	Step count, physical activity time (min/week), MET/min/week spent in walking, spent calories/week.	None.
Moniruzzaman (2015, Canada)	Examine the relationship between trip distance and socio-demographic attributes and accessibility features of lower-income older adults in Metro Vancouver.	Total walking, wfrequency, total physical activity, leisure time, physical activity.	None.
Rijken (2010, Netherlands)	Examine the relationship between pet ownership and health-related outcomes (physical activity, social contacts, and feelings of loneliness).	Walking for exercise, non-exercise-related walking, vigorous activity (frequency and time).	None.
Rogers (1993, USA)	Investigate how dog ownership affects conversations while walking, exercise levels, and social and psychological functioning.	Number and duration of walks per day.	Conversations duration, and content.
Scheibeck (2011, Austria)	Examine the human–dog relationship among older adults.	Average daily accelerometer step-counts and time spent in different physical activity levels.	None.
Shibata (2012, Japan)	Examine the association between dog ownership, dog walking, and physical activity in older Japanese adults.	Healthy living (y/n: at least moderately physically active for 30 min on at least 5 days per week).	Frequency of social contacts with friends or acquaintances, and neighbors.<br>UCLA Loneliness Scale.
Taniguchi (2018, Japan)	Examine physical function, physical activity, social function, and psychological function of a population of community-dwelling older Japanese dog and cat owners.	Metabolic equivalents (METs).	Questions on perception and use of neighborhood.
Thorpe (2006a, USA)	Determine whether dog owners are more likely to engage in physical activity than non-dog pet or non–pet owners.	Total physical activity (min/wk; metabolic equivalent-hr/wk); sedentary time; total hours spent sleeping or lying down.	None.
Thorpe (2006b, USA)	Examine dog walking among dog owners and the relationship between the walking behavior of dog owners and non-dog owners over 3 years.	Trip length in km for walk, car and transit modes.	None.
Wu (2017, UK)	Investigate the role of dog ownership and walking as a means of supporting the maintenance of physical activity in older adults during periods of inclement weather.	Daily physical activity (counts per minute) and minutes of sedentary behavior	None.

Supplementary Table 2. Explanatory variables analyzed.

Studies	Dog-related variables	Environmental variables	Covariates
Arbillaga-Etxarri (2017, Spain)	Having a dog at home (y/n); Walking the dog (y/n).	Neighborhood deprivation, residential greenness, residential proximity to green or blue spaces.	Education, marital status, working status, occupation, household size, smoking status, dyspnea, quality of life, anxiety and depression.
Carr (2021, USA)	Frequency of dog walking (score).	None.	COVID-19 related exposures, other pet, age, education, sex, marital status, employment, self-rated health, social support and hassles from friends, impact of stressful events.
Chen (2020, China)	Frequency of dog walking (number).	Age- and dog-friendly space for leisure.	Age, occupation.
Curl (2017, USA)	Dog ownership (y/n); Dog walking (y/n), Frequency of dog walking (score); Dog walking time (min); Pet bonding (index).	None.	Age, household income, gender, ethnicity, years of education, and marital status.
Curl (2020, USA)	Owing a dog (y/n). Number of times the person walked the dog(s); average of time the dog(s) was walked each time. Pet Attachment Scale.	None.	Education, age, marital status, household income, and self-rated health.
Dall (2017, The Netherlands)	Dog ownership (y/n); demographics (age, type, size, gender, and length of ownership). Details in caring for the dog(s).	None.	Age, gender, ethnicity, socio-economic status, cat ownership.
Dzhambov (2017, Bulgaria)	Dog size.	Territory, population; green spaces, park dog area and quality. Perceived access, complaints, frequency of dog-waste disposal, preference of isolated parks.	Age, sex, ethnicity, education, marital status, socio-economic class, perceived health status.
Feng (2014, UK)	Pet ownership.	Nearby green space, urban or rural, weather condition (rain, temperatures).	Household income, marital status, education, health status, Hospital Anxiety and Depression Score (depression), social capital.
Friedmann (2020, USA)	10-year pet-ownership history; Health and Retirement Study; Lexington Attachment to Pets Scale.	None.	Sex, education, income, marital status, household, other pet ownership, past pet ownership, health status, cognitive function, psychological adaptation, anxiety.
Garcia (2015, USA)	Dog ownership (y/n).	None.	Age, race, education, income, household, smoking status, BMI, history of chronic diseases, self-reported physical function.
Gretebeck (2013, USA)	Dog ownership (y/n), reason for walking (walk dog, fun, exercise, getting to work or store, instructed by provider).	None.	Age, gender, functional ability, BMI. Attitude toward performing physical activity for 30 minutes 3 days/week. Perceived behavioral control.
Harris (2009, UK)	Dog walking (y/n).	None.	Age, sex, pedometer use and household clustering, disability score, health, BMI, exercise self-efficacy, control over exercise.
Hui Gan (2020, Australia)	Relationships with pets.	None.	Age, gender, home help, household, number, type and length of pets owned.
Janevic (2020, USA)	Pet ownership (dog or cat).	None.	Age, sex, ethnicity, education, health, average pain intensity.
Koohsari (2021, Japan)	Dog ownership, dog walking status.	None.	Age, sex, education, marital status, length of residence at the current address.
Mein (2018, UK)	Dog ownership (y/n), Attachment to pet (Likert scale).	None.	Cognitive status, age, marital status, occupation, retirement, social activities, illness.
Mičkova (2019, Czech Republic)	Dog ownership (y/n).	None.	None.
Moniruzzaman (2015, Canada)	Dog ownership (y/n).	Neighborhood perceptions.	Age, sex, education, household, ethnicity, driver's license, mobility aids, fallen in past 6 months, confidence in walking.

Rijken (2010, The Netherlands)	Dog ownership (y/n).	None.	Age, gender, education, marital status, health status.
Rogers (1993, USA)	Dog ownership (y/n).	None.	None.
Scheibeck (2011, Austria)	None.	Having a backyard (y/n).	Age, sex.
Shibata (2012, Japan)	Dog ownership (y/n), dog walking (y/n), number of times per day; for how long (min/day); number of days per week.	None.	Gender, age, city, education; employment status; living with family or others; and self-rated health, BMI.
Taniguchi (2018, Japan)	Dog ownership (y/n).	None.	Sex, age, household, marital status, education, income; history of chronic diseases, pain, fall; sleep; Tokyo Metropolitan Institute of Gerontology Index of Competence.
Thorpe (2006a, USA)	Dog ownership (y/n).	Site (Memphis or Pittsburgh).	Age, sex, race, marital status, education, income, employment, currently smoke, household, health condition, past falls, depressive symptoms, BMI.
Thorpe (2006b, USA)	Dog ownership (y/n), frequency of dog walking.	Site (Memphis or Pittsburgh).	Age, sex, race, marital status, education, income, employment, currently smoke, household, health condition, past falls, depressive symptoms, BMI.
Wu (2017, UK)	Dog ownership (y/n), frequency of dog walking.	Temperature, precipitation, day length.	Age, sex, education, health status.
